# Cell Wall Layer Induced in Xylem Fibers of Flax Upon Gravistimulation Is Similar to Constitutively Formed Cell Walls of Bast Fibers

**DOI:** 10.3389/fpls.2021.660375

**Published:** 2021-04-06

**Authors:** Anna Petrova, Liudmila Kozlova, Oleg Gorshkov, Alsu Nazipova, Marina Ageeva, Tatyana Gorshkova

**Affiliations:** ^1^Laboratory of Plant Cell Growth Mechanisms, Kazan Institute of Biochemistry and Biophysics, Federal Research Center Kazan Scientific Center of Russian Academy of Sciences, Kazan, Russia; ^2^Microscopy Cabinet, Kazan Institute of Biochemistry and Biophysics, Federal Research Center Kazan Scientific Center of Russian Academy of Sciences, Kazan, Russia

**Keywords:** flax fibers, gravitropic response, atomic force microscopy, biomechanical properties, tertiary cell wall, transcriptome, rhamnogalacturonan I, rhamnosyltransferases

## Abstract

In the fibers of many plant species after the formation of secondary cell walls, cellulose-enriched cell wall layers (often named G-layers or tertiary cell walls) are deposited which are important in many physiological situations. Flax (*Linum usitatissimum* L.) phloem fibers constitutively develop tertiary cell walls during normal plant growth. During the gravitropic response after plant inclination, the deposition of a cellulose-enriched cell wall layer is induced in xylem fibers on one side of the stem, providing a system similar to that of tension wood in angiosperm trees. Atomic force microscopy (AFM), immunochemistry, and transcriptomic analyses demonstrated that the G-layer induced in flax xylem fibers was similar to the constitutively formed tertiary cell wall of bast (phloem) fibers but different from the secondary cell wall. The tertiary cell walls, independent of tissue of origin and inducibility, were twice as stiff as the secondary cell walls. In the gravitropic response, the tertiary cell wall deposition rate in xylem was higher than that of the secondary cell wall. Rhamnogalacturonan I (RG-I) with galactan side chains was a prominent component in cellulose-rich layers of both phloem and xylem flax fibers. Transcriptomic events underlying G-layer deposition in phloem and xylem fibers had much in common. At the induction of tertiary cell wall deposition, several genes for rhamnosyltransferases of the GT106 family were activated in xylem samples. The same genes were expressed in the isolated phloem fibers depositing the tertiary cell wall. The comparison of transcriptomes in fibers with both inducible and constitutive tertiary cell wall deposition and xylem tissues that formed the secondary cell walls is an effective system that revealed important molecular players involved in the formation of cellulose-enriched cell walls.

## Introduction

The plant cell wall is a complex polysaccharide-based structure, whose composition and architecture depend on plant species, cell function, stage of development, and environmental factors. In the fibers of many plant species, cellulose-enriched cell wall layers (often named gelatinous layers or G-layers) are deposited after the formation of secondary cell walls that are important in many physiological situations (Gorshkova et al., 2012, 2018). These layers are deposited only in fibers, although these cells may be derived from both primary and secondary meristems and belong to both xylem and phloem in various plant organs. Examples include the fibers of roots and hypocotyls of geophytes (Fisher, 2008; Schreiber et al., 2010; Tomlinson et al., 2014), aerial roots (Zimmermann et al., 1968; Tomlinson et al., 2014), vine stems (Fisher and Blanco, 2014), and spines and cladode junction regions of cactuses (Bobich and Nobel, 2002). The best-studied examples are the fibers of tension wood (Côté et al., 1969; Gorshkova et al., 2015; Gritsch et al., 2015; Guedes et al., 2017) and the bast fibers of fiber crops (reviewed in Gorshkova et al., 2012; Gorshkova et al., 2018). In tension wood of trees, such fiber cell wall layers are induced in negative gravitropic reactions and are deposited asymmetrically in fibers from one side of inclined stems (Fahn, 1990; Groover, 2016). Cellulose-enriched cell wall layers of bast fibers emerge during constitutive fiber development and are in all fibers around the stem circumference (Esau, 1965). Thus, the regulation of such cell wall layer deposition may have distinct features. However, in fibers of different origin, compositions and architectures of the cellulose-enriched cell wall layers are very similar. These layers have an exceedingly high content of cellulose (Norberg and Meier, 1966) and close to axial orientation of cellulose microfibrils (Clair et al., 2011). Xylan is absent (Gorshkova et al., 2015; Chernova et al., 2018; Behr et al., 2019), as is lignin (Love et al., 1994; Kaku et al., 2009; Gorshkova et al., 2012). The latter, however, can sometimes be deposited at a late stage of fiber maturation (Roussel and Clair, 2015). The major polysaccharide of the cell wall matrix is rhamnogalacturonan I (RG-I) with side chains of β-1,4-galactans (Gorshkova et al., 2010, 2015; Guedes et al., 2017).

Despite such similarities, fibers of tension wood and bast fibers are usually studied separately, with different scientific schools using different approaches and terminology (reviewed in Clair et al., 2018; Gorshkova et al., 2018). Cellulose-enriched cell wall layers of tension wood fibers are called G-layers of secondary cell walls (Clair et al., 2018). Similar layers in bast fibers are called tertiary cell walls to emphasize the major differences from secondary cell walls in cell wall composition, architecture, mode of formation, and function (Gorshkova et al., 2018). The name is based on the sequence of deposition: a fiber first deposits the primary cell wall, then the secondary cell wall, and, only then, the tertiary cell wall (Gorshkova et al., 2012). The tertiary cell wall is found only in fibers and provides tissue tension (Alméras et al., 2020), whereas the secondary cell wall fixes cell shape after cell enlargement is completed. The secondary cell wall is in all fibers of all plant species, whereas the tertiary cell wall, although widespread in fibers of different origin, is not necessarily in all fibers of the same plant or in all plant species (Gorshkova et al., 2012). The tertiary cell wall is characterized by intensive post-deposition remodeling in which the newly formed portions of cell wall material are later transformed into mature ones. The newly deposited and mature layers of the tertiary cell wall can be distinguished under microscopy and were historically named as Gn- and G-layers, respectively (Gorshkova et al., 2004).

Flax (*Linum usitatissimum* L.) and many other angiosperms have fibers in both the phloem and xylem parts of the stem (Esau, 1965). Flax phloem fibers constitutively develop thick tertiary cell walls during normal plant growth, whereas xylem fibers of the flax stem deposit only secondary cell walls (Gorshkova et al., 2012). However, in the gravitropic response after plant inclination, the formation of cellulose-enriched cell walls is induced in xylem fibers on the upper side of the stem (Ibragimova et al., 2017), resembling the tension wood formation. Flax xylem and phloem fibers differ in their origin and morphology (Esau, 1965). In flax, primary phloem fibers originate only from the procambium close to the apical meristem, whereas most xylem fibers in the stem originate from the cambium (Esau, 1965; Fahn, 1990). Although primary phloem fibers are the longest plant cells, reaching many centimeters, the length of secondary xylem fibers is less than a millimeter (Bourmaud et al., 2018).

A comparison of the properties of cellulose-enriched cell walls formed in phloem and xylem fibers of the same plant may reveal similarities and differences between constitutively and inducibly formed cellulose-enriched cell wall layers in fibers that are different in origin and morphology. Therefore, atomic force microscopy (AFM), immunochemistry, and the expression of marker genes were used to compare cell walls formed in phloem and xylem fibers of flax. The study showed that the cellulose-enriched cell wall layer induced in flax xylem fibers under gravistimulation was similar to the constitutively formed tertiary cell wall of bast fibers but very different from the secondary cell wall.

## Materials and Methods

### Plant Material and Gravistimulation

**FIGURE 1:**
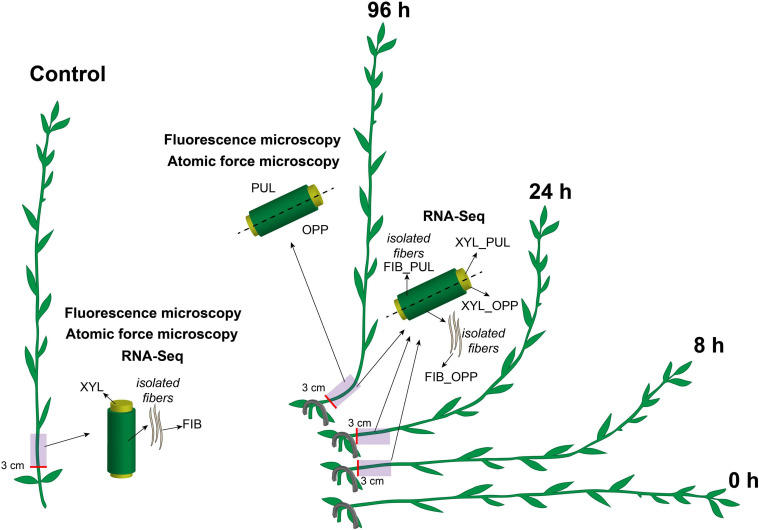
Schematic of the experimental design. Flax plants were inclined and stapled to the soil at the level of the cotyledons. Gravitropic response developed over 96 h. Five-cm-long stem segments beginning at 3 cm above the cotyledons were used in the analyses. The upper part of the gravitropic bend was designated as the pulling side (PUL) and the lower part as the opposite side (OPP). Phloem and xylem (XYL) parts of the stem were collected separately for RNA-Seq in the control and after 8, 24, and 96 h of gravitropic response. Phloem parts were washed several times in ethanol in a mortar with gentle pestling to isolate phloem fibers (FIB). All samples for fluorescence microscopy and atomic force microscopy were collected 96 h after gravistimulation. Control samples were taken from the non-inclined plants, at the same stem level and at the same time as for the inclined plants.

Flax plants (*L. usitatissimum* L. “Mogilevsky”) were grown in boxes with a 50-cm soil layer under natural light with daily watering. Forty-day-old plants (approximately 35 cm in height) were stapled to the soil at the cotyledon level to orient them horizontally. The gravitropic response was developed for 96 h during which plants returned to a vertical position by forming a gravitropic bend in the lower part of the stem (Figure 1). The gravitropic bend region (an approximately 5-cm-long segment beginning 3 cm above the cotyledons) and the corresponding part of control plants (which were not inclined) were studied. The upper (concave) part of the gravitropic bend was designated as the pulling side and the lower (convex) part as the opposite side (Figure 1). The phloem and xylem parts of the stem were collected separately for RNA-Seq in the control and after 8, 24, and 96 h of gravitropic response. Fiber-enriched stem peels represented phloem parts of the stem. They were washed several times in 80% (w/v) ethanol in a mortar with gentle pestling to isolate phloem fibers (sample FIB in RNA-Seq). Undivided stem parts in the control and stem regions divided into pulling and opposite sides after 96 h of gravistimulation were collected for fluorescence microscopy and AFM.

### Sample Preparation for Fluorescent and Atomic Force Microscopy

A 1-cm-long fragment was cut from the initially collected 5-cm-long stem segments (see above). Segments of gravistimulated flax were divided into pulling and opposite sides. Control stems were also cut in halves vertically to provide better solution permeability. Samples were fixed overnight in 4% (w/v) paraformaldehyde solution in 0.2 M phosphate-buffered saline (PBS, pH 7.4). For proper AFM investigation, stem fragments needed to be oriented vertically in the embedding capsule. To achieve this goal, stem fragments were first embedded in 3% (w/w) low-melting point agarose. Then, agarose blocks with samples were dehydrated in a graded aqueous ethanol series (80:20, 60:40, 40:60, 20:80, and 0:100) and progressively infiltrated with London Resin White (LRW, EMS, Hatfield, PA, United States) in a graded acetone LRW series (80:20, 60:40, 40:60, 20:80, and 0:100). During this passage, the samples were stored at +4°C to avoid polymerization. Samples in 100% resin were kept overnight at −20°C and then for several h at room temperature. The heat curing was performed in flat-bottom Beem capsules (EMS, Hatfield, PA, United States) at 60°C for 24 h.

### Immunohistochemistry

Thin sections (700 nm thickness) for immunohistochemistry were prepared using a glass knife on an LKB8800 ultramicrotome (LKB Instruments, Bromma, Sweden) and collected on silane-coated microscope slides (EMS, Hatfield, PA, United States). Immunohistochemical analysis of cell wall polymers was performed using LM5 and LM11 (Leeds University, Leeds, United Kingdom) antibodies. For immunolocalization, the sections were (1) blocked with 0.2 M PBS (pH 7.4) containing 2% (w/v) bovine serum albumin for 30 min at room temperature; (2) incubated with one of the primary monoclonal antibodies diluted 1:5 for 1.5 h at room temperature and then washed three times with PBS; and (3) incubated with secondary anti-rat IgG linked to fluorescein isothiocyanate (FITC, Sigma-Aldrich, St. Louis, MO, United States) diluted 1:100 in PBS (0.2 M, pH 7.4) for 1 h at room temperature in the dark. The primary antibody treatment was omitted for the negative controls. After the labeling reaction, the sections were washed four times in PBS and twice in water. Sections were observed using a Leica DM1000 epifluorescence microscope (Leica Biosystems, Wetzlar, Germany) fitted with a mercury lamp. Sections were observed under epifluorescence settings with the filter cube (excitation filter 460–500 nm, barrier filter 512–542 nm). Exposure time was maintained constant. The fluorescence intensity was measured on high-magnification images using ImageJ2 Fiji software^1^. After background removal by “rolling ball” function, the mean signal intensity was determined in the square of fixed size placed on the particular cell wall layer.

### Atomic Force Microscopy

Resin blocks with embedded stem parts that remained after sectioning for immunochemical analysis were additionally polished by diamond knife (EMS) on an LKB8800 ultramicrotome (LKB Instruments). AFM was performed at room temperature and constant humidity using an NTEGRA Prima (NT-MDT, Zelenograd, Russia) microscope. The AFM HybriD mode was used to obtain the stiffness and elasticity maps using HA_HR tips (NT-MDT) with a typical resonance frequency of 380 kHz, average spring constant of 34 N m^–1^, and apex radius of 10 nm. The thermal tune procedure was performed for each new cantilever to determine its unique spring constant. Deflection sensitivity was determined at room temperature on a fresh cover glass for each new cantilever, between samples, and every time after laser adjustment. Scanning was conducted at a speed of 5 s per line in both forward and backward directions. The typical scanning area was 70 × 70 μm with 512 × 512 point resolution. Force-indentation curves devoid of artifacts were selected manually from the elasticity map and fitted to a Derjaguin–Muller–Toporov (DMT) model of contact between a sphere and a half-space. The reliability of obtained data was checked by the apparent elastic modulus of LRW resin, which had to be between 3 and 5 GPa, as shown by Arnould et al. (2017) using a similar technique. Measurements of cell wall thickness were conducted on stiffness maps using the line tool of Nova Px 3.4 software (NT-MDT).

### RNA-Seq Data Processing

The high-quality reads of 28 RNA-Seq data sets from the stem samples of non-inclined control and gravistimulated plants after 8, 24, and 96 h of gravitropic response (Figure 1) were obtained, deposited as BioProject PRJNA631357 in the Sequence Read Archive^2^, verified by PCR, and partially described previously (Mokshina et al., 2017, 2018, 2020; Gorshkov et al., 2018). In this paper, the names of the samples were simplified compared with those of deposited ones. sXYLb in the database corresponds to XYL in the current paper and tFIBb to FIB.

The bioinformatic analysis was performed according to the algorithm presented in Mokshina et al. (2020). Briefly, the alignment of reads to the flax genome (Wang et al., 2012), transcriptomic assembly, and analysis of differentially expressed genes were performed using HISAT2, StringTie (Pertea et al., 2016), and the R package DESeq2 (Love et al., 2015). In the pre-filtered data set of 31,351 genes, those with normalized counts of total gene reads (TGR) more than or equal to 16 in at least one sample were considered to be expressed, according to the recommendation of the SEQC/MAQC-III Consortium (2014). The mRNAs with twofold changes in expression and *p*_adjusted_ (adjusted *p*-value) < 0.01 were identified in DESeq2 as differentially expressed genes. The pairwise comparison with control samples was conducted at each time point according to the stem fragment (pulling or opposite side) and tissue type (phloem fibers or xylem). The search for the closest *Populus* homologs of the target flax genes was performed using the program BLAST+ (Camacho et al., 2009) and the Plant Comparative Genomics portal, Phytozome v12^3^.

### Phylogenetic Analysis

The flax genes of the glycosyltransferase (GT) 106 protein family were identified by the PF10250 Pfam domain in protein sequences of the flax genome using HMMer 3.3 software^4^ and a local BLAST search implemented in BioEdit 7.0.5 software (Hall, 1999) with *AtRRT1* (*At5g15740*) as a query. The Pfam domain profile was obtained from the Pfam 32.0 database^5^ (El-Gebali et al., 2019), and the flax genome was downloaded from the Phytozome v12.1 database^6^ (Goodstein et al., 2012). The protein sequences of deduced flax GT106 genes were additionally checked for the characteristic IPR024709 domain in the InterProScan tool of the InterPro database^7^ (Mitchell et al., 2019). Sequences that did not possess that domain were excluded from further analysis. The *Arabidopsis thaliana* GT106 member gene list by Takenaka et al. (2018) was annotated according to the Uniprot database (release 2020_05)^8^ (Bateman et al., 2019). The obtained plant protein sequences were aligned with one another in the web-based service Clustal Omega^9^ (Madeira et al., 2019). The alignment was subjected to a maximum-likelihood phylogenetic analysis, which was performed using IQTREE1.6.9 software (Nguyen et al., 2015). The best-fit model of sequence evolution was selected using Bayesian Information Criterion implemented in ModelFinder (IQTREE1.6.9) (Kalyaanamoorthy et al., 2017). The ultrafast bootstrap branch support (Minh et al., 2013) with 10,000 replicates was used to construct the dendrogram (values less than 95 were not significant). The unrooted tree was visualized using the web-based service iTOL 5.3^10^ (Letunic and Bork, 2019) and corrected in Adobe Illustrator CC 2017.

### Statistics

Four biological replicates of each sample were examined for cell wall mechanical properties using AFM. Four biological replicates for each antibody were used in the immunocytochemical analysis. Mean values with standard deviations among biological replicates are presented. Mean separation was performed by ANOVA followed by Tukey’s test at α = 0.01, using the SPSS software (v 21, IBM Corp., Armonk, NY, United States).

## Results

Flax gravistimulation was conducted as described previously (Ibragimova et al., 2017, 2020; Gorshkov et al., 2018; Figure 1). Plants returned to the vertical position 96 h after the inclination. The most pronounced gravitropic bend was formed in the stem region above the cotyledons. The upper (concave) part of the gravitropic bend was designated as the pulling side and the lower part (convex) as the opposite side (Figure 1).

### Immunohistochemistry

**FIGURE 2:**
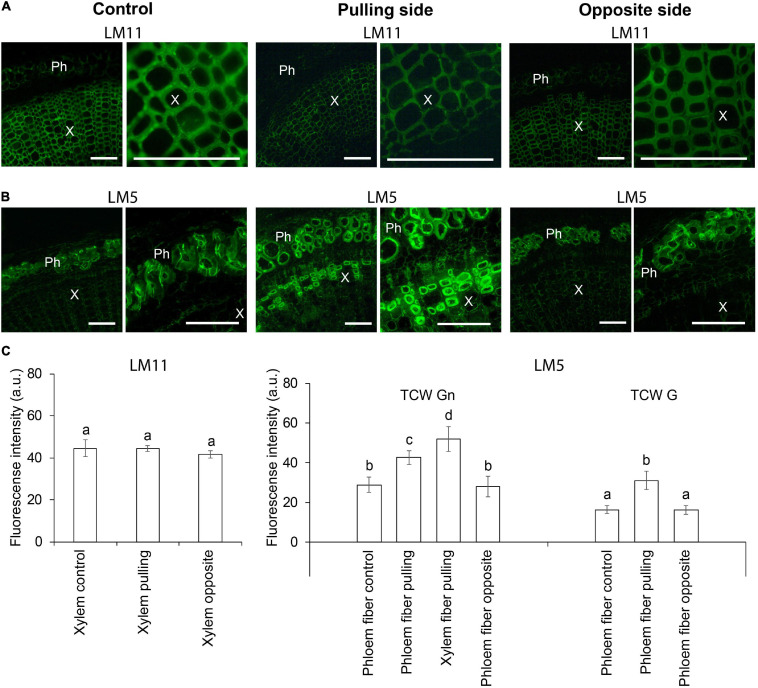
Fluorescence micrographs of flax stem cross sections immunolabeled by **(A)** LM11 (xylan) and **(B)** LM5 (1,4-galactan) antibodies in control plants and after 4 days (96 h) of gravistimulation. Bars are 100 μm. Ph, phloem; X, xylem. **(C)** Mean fluorescence intensity of different cell walls. TCW, tertiary cell wall; G and Gn, G and Gn layers. Values are the mean ± SD. Different letters above the bars indicate a significant difference according to one-way ANOVA followed by Tukey’s test at α = 0.01.

Immunohistochemical analysis was performed to clarify the nature of thickened cell walls in different stem tissues in response to the gravistimulation of flax plants. Xylan is a typical component of secondary cell walls, whereas β-1,4-galactan is characteristic of tertiary cell walls (Gorshkova et al., 2018). Thus, LM11 and LM5 antibodies recognizing 1,4-β-D-xylan/arabinoxylan and 1,4-β-D-galactan, respectively, were used on cross sections of control and 96-h gravistimulated flax plants (Figure 1). Consistent with previous observations (McCartney et al., 2005; Chernova et al., 2020), the LM11 antibody was heavily bound to xylem cell walls in control plants (Figure 2A), whereas in phloem fibers, xylan epitopes were restricted to the thin outer cell wall layer and were not present in the thick tertiary cell walls. The LM11 antibody bound to xylem cell walls on both the pulling and opposite sides of the gravitropic bend. However, the labeled cell walls in the xylem of the pulling side appeared thinner than those of the opposite side and control plants (Figure 2A).

The LM5 antibody was detected in the thickened cell walls of the phloem fibers in control and pulling- and opposite-side samples (Figure 2B), confirming their tertiary nature. The labeling of phloem fibers on the pulling side was more intense than that in the control or opposite-side samples (Figure 2C). This difference might be due to a change in the ratio of G- and Gn-layers in the tertiary cell wall of the different phloem fibers. On the pulling stem side, the LM5 antibody bound to the thickened cell walls of some xylem fibers (Figure 2B). This result indicated that those fibers might have switched to deposition of tertiary cell walls instead of secondary ones as a result of gravistimulation.

### Atomic Force Microscopy of Cell Walls

**FIGURE 3:**
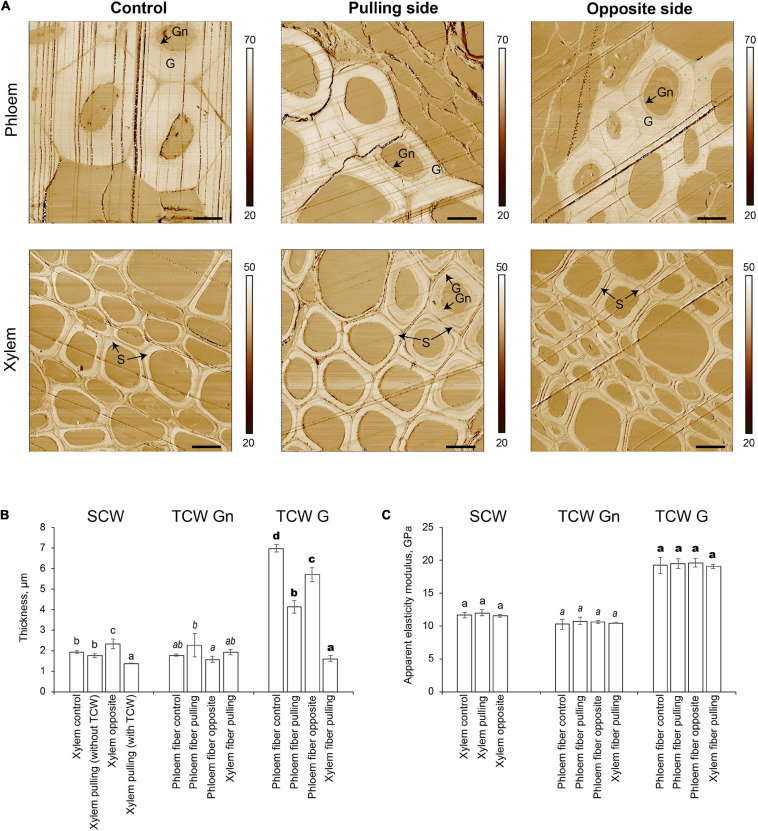
Atomic force microscopy of fiber cell walls in control and gravistimulated flax plants 96 h after stem inclination. **(A)** Stiffness (N/m) maps of phloem (top) and xylem (bottom) fibers in control and gravistimulated flax plants. One representative image for each variant is shown. Stiffness scales are on the right side of each map. Bars are 10 μm. G- and Gn-layers of tertiary cell walls in phloem and similar structures in the cell walls of xylem fibers on the pulling side are indicated. S indicates secondary cell walls. Parallel lines on each image are scratches left by the knife. **(B)** Thickness (μm) and **(C)** Apparent elasticity modulus (GPa) of different cell wall layers. SCW, secondary cell walls; TCW, tertiary cell walls. Values are the mean ± SD. Different letters above the bars indicate a significant difference according to one-way ANOVA followed by Tukey’s test at α = 0.01 (individual comparisons are shown by regular, italic, and bold-type fonts of these letters).

The two distinct layers of tertiary cell walls, G and Gn, were easily observed in some phloem fibers on stiffness maps (Figure 3A). The G-layers appeared denser than the Gn-layers. The stiffness of G-layers was higher than that of Gn layers, as determined by the lighter tone of the former than the latter on stiffness maps (Figure 3A). Phloem fibers with G- and Gn-layers were observed in control plants and on both pulling and opposite sides of gravistimulated plants. Similar structures appeared in some xylem fibers on the pulling side. The AFM results correlated well with the LM5 labeling and further confirmed the tertiary nature of the thickened cell walls.

The thickness of different cell wall layers in different tissues was measured in the stiffness maps (Figure 3B). In control plants, the thickness of the secondary cell wall layer of xylem cells was 1.92 ± 0.07 μm. In gravistimulated plants, xylem cells on the opposite side had the thickest secondary cell walls (2.33 ± 0.24 μm, Figure 3B). The secondary cell wall thickness between xylem cells on the pulling side of the stem was different depending on whether tertiary cell walls were present. In cells without a tertiary cell wall layer, the secondary cell wall thickness was similar to that of control plants, whereas with a tertiary cell wall, the secondary cell walls of xylem cells on the pulling side were significantly thinner (1.36 ± 0.01 μm) than those of other xylem cells. With this value considered as a zero point for secondary cell wall formation, xylem fibers in control plants and those that did not begin to develop G-layers deposited 0.39–0.56 μm of secondary cell walls over 96 h. Xylem cells on the opposite side of the stem accumulated 0.97 μm of cell wall thickness in the same period.

Different layers of tertiary cell walls (G and Gn) were measured separately. The Gn-layers in phloem fibers and the xylem cells on the pulling side of inclined plants were 2.25 ± 0.57 and 1.92 ± 0.07 μm thick, respectively. The G-layers of phloem fibers were the thickest in the control (6.98 ± 0.18 μm). Phloem fibers on both pulling and opposite sides of gravistimulated plants had thinner G-layers than that of the control; this effect was especially pronounced in fibers on the pulling side (Figure 3B). Among all phloem fibers, the lowest G/Gn ratio was for those on the pulling side. This result might explain the difference in the intensity of their LM5 labeling (Figure 2), because Gn is more intensively labeled with this antibody (Gorshkova et al., 2004). Xylem fibers on the pulling side deposited 1.61 ± 0.14 μm of G-layer in the gravitropic response.

Apparent elasticity moduli of different cell wall layers deposited by cells of different tissues were also measured by AFM (Figure 3C). Secondary cell walls of xylem had an apparent elasticity modulus of 11.5–12.0 GPa, whether or not the plant was inclined. Similarly, there was no difference between the mechanical properties of Gn-layers, with no dependence on the plant state or the tissue that produced the cell walls (Figure 3C). The apparent elasticity modulus of G-layers (19.1–19.6 GPa) was higher than that of either secondary cell walls or Gn-layers. Values were similar in phloem fibers of control and pulling and opposite sides of gravistimulated plants, as well as for G-layers developed by xylem cells on the pulling side.

### Genes Specifically Expressed in the Xylem of Gravistimulated Plants

The induction from zero tertiary cell wall deposition in xylem fibers from the pulling stem side in the gravitropic response provided the basis to reveal the genes involved. Gene expression was evaluated by comparing transcriptomes in xylem stem parts from the pulling stem side with those in the initial state in the non-inclined control and the opposite stem side. In the pairwise comparison of xylem samples from the gravistimulated and control flax stems, 5,582 upregulated genes were identified, of which 222 genes were characteristic only for the pulling side of the stem and were activated at least in one of the analyzed time-points after gravistimulation (8, 24, and 96 h).

Ten genes had significantly higher expression in xylem cells of the pulling stem side than in those of both the opposite side and control plants at all stages of gravitropic response (Fold change ≥ 2, *p*_adjusted_ < 0.01) (Table 1). Four of the genes encoded fasciclin-like arabinogalactan (FLA) proteins (*Lus10036113*, *Lus10002985*, *Lus10036114*, and *Lus10002986*), and three were annotated as O-fucosyltransferases (*Lus10035540*, *Lus10007975*, and *Lus10013503*). The other three genes were annotated as a protein kinase (*Lus10016382*), a protein of unknown function, DUF707 (*Lus10032090*), and a cytochrome P450, CYP76C6 (*Lus10006323*). The expression of these genes (except *Lus10006323*) also increased in the phloem fibers of the pulling side compared with that of control phloem fibers.

**TABLE 1:** Flax genes continuously upregulated (fold change ≥ 2, *p*_adjusted_ < 0.01) in the pulling-side xylem but not in the opposite side of inclined plants in the comparison with control xylem.

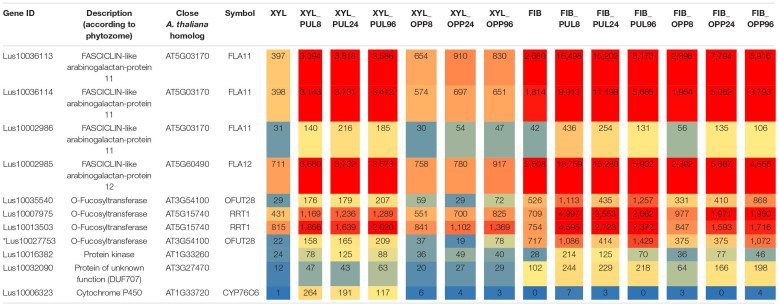

Values are total gene reads (TGR). XYL, xylem; FIB, isolated phloem fibers; PUL, pulling side of the stem; OPP, opposite side of the stem; 8, 24, and 96 h after plant inclination. Blue–yellow–red heat map indicates the values of TGR on a *min* (blue) to *max* (red) color gradient. *Lus10027753 was also upregulated in the opposite-side xylem.

**FIGURE 4:**
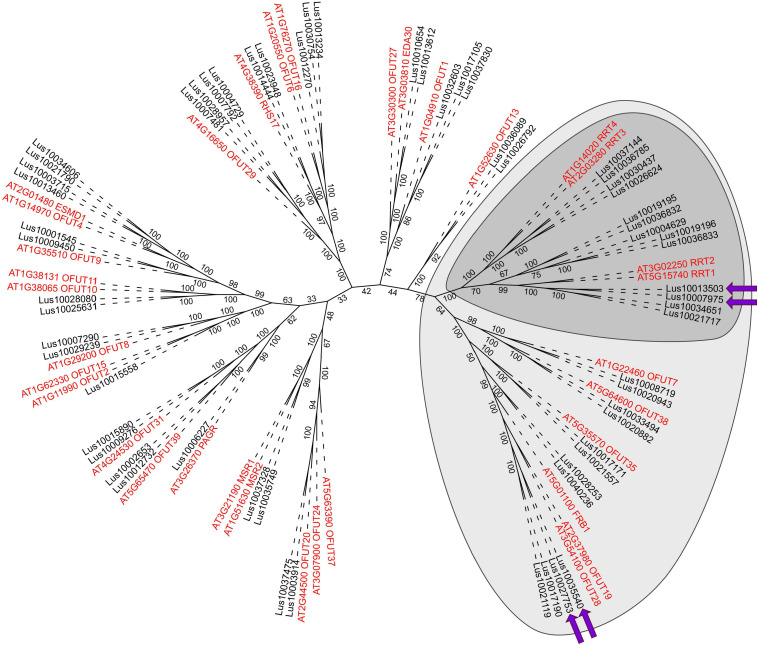
Maximum-likelihood phylogenetic dendrogram of plant GT106 protein family members (with PF10250 and IPR024709 domains) of flax (black) and *Arabidopsis thaliana* (red). Dark gray confines the RG-I:rhamnosyltransferase (RRT) clade, according to Takenaka et al. (2018), and light gray indicates the RRT clade size, according to Wachananawat et al. (2020). Purple arrows show genes upregulated in the pulling side xylem during gravitropic response. The *A. thaliana* gene names are according to the annotation of the Uniprot database^8^ (Bateman et al., 2019). Numbers indicate the ultrafast bootstrap support values; branch lengths were ignored.

The putative O-fucosyltransferases encoded by the upregulated genes had the PF10250 domain in their predicted amino acid sequences and thus belong to the GT106 protein family (Takenaka et al., 2018). Four members (of 34 total in *A. thaliana*) in this family have been characterized as RG-I:rhamnosyltransferases (RRTs) involved in the formation of the RG-I backbone (EC 2.4.1.351) (Takenaka et al., 2018). To establish the relations between the flax genes upregulated during the gravitropic response and the characterized members of GT106, the phylogenetic tree of that family was constructed (Figure 4).

Several gene clades formed the dendrogram of the GT106 members of flax and *A. thaliana*. Flax had twice as many GT106 members as *Arabidopsis* (61 vs. 34), which may indicate the specific physiological importance of reactions catalyzed by those enzymes for flax. According to Takenaka et al. (2018), the RRT clade consists of four *Arabidopsis* genes encoding RRT1, RRT2, RRT3, and RRT4, whereas the clade included 13 flax homologs (Figure 4). Four of the flax genes (including the upregulated *Lus10007975* and *Lus10013503*) were in the same clade as *RRT1* (*At5g15740*) and *RRT2* (*At3g02250*) (Figure 4), suggesting similar catalytic functions.

The upregulated *Lus10035540* and three other flax genes were in another clade of the GT106 tree with *At3g54100*, *At3g37980*, and *At5g01100* (FRIABLE1, FRB1) (Figure 4). Of these genes, *Lus10027753* was also upregulated in xylem of the pulling side (Table 1, gene shown by asterisk) but did not fully meet the criteria, because at 96 h after stem inclination, its upregulation was also detected in the opposite-side xylem. A recent study of GT106 members in *Marchantia polymorpha* also suggests that this clade contains RG-I:rhamnosyltransferases (Wachananawat et al., 2020; Figure 4). All four genes in the GT106 family that were upregulated in the xylem of the pulling stem side were actively expressed in all samples of phloem fibers (Table 1).

## Discussion

### Nanomechanical Properties of Cell Walls Depend on Whether They Are Tertiary or Secondary but Not on the Tissue That Produces Them and Remain the Same in the Flax Gravitropic Response

AFM is a technique widely used to examine the mechanical properties of cell walls. The G and Gn apparent elasticity modulus values determined using AFM were in the same ranges as those shown previously for flax phloem fibers (Arnould et al., 2017; Goudenhooft et al., 2018). Similarly, the apparent modulus of secondary cell walls in hemp xylem determined using AFM (Coste et al., 2020) was comparable with that in flax (Figure 3C).

In the development of flax phloem fibers, there is a gradual transition from the newly deposited Gn-layer to the denser G-layer, which is accompanied by changes in their composition and architecture (Gorshkova et al., 2004, 2018). A corresponding increase in stiffness in the Gn to G transition reported previously (Goudenhooft et al., 2018) was confirmed in the current study (Figure 3C). Apparent elastic moduli of these layers remained constant during flax phloem fiber development, independent of the Gn/G ratio (Goudenhooft et al., 2018). The gravitropic reaction of flax also induced a difference in the ratio of G- and Gn-layer thickness between different sides of the plant and control plants (Figure 3B). However, the nanoscale properties of these layers themselves remained the same (Figure 3C). This observation is in contrast to that of Goudenhooft et al. (2019b) who observed changes in the mechanical performance of flax fibers with gravistimulation. However, tensile tests in that work were performed at the level of individual fibers, which can be strongly affected by major morphological changes occurring in phloem fibers of gravistimulated flax (Ibragimova et al., 2017), while their nanoscale properties remain constant. Moreover, in the current study, the nanomechanical properties of tertiary cell walls were independent of the tissue that produced them (Figure 3C). Thus, an intriguing possibility emerges of determining the cell wall nature (secondary or tertiary) by its apparent Young’s modulus.

### Xylem Fibers Deposit Tertiary Cell Wall in the Gravitropic Reaction in Flax

Tertiary cell walls have several distinguishing features (Gorshkova et al., 2018). The RG-I with long galactan side chains is crucial in forming specific tertiary cell wall architecture (Gorshkova et al., 2010, 2018; Guedes et al., 2017). Either a galactan or RG-I backbone can be labeled in tertiary cell walls using LM5 or RU2 antibodies, respectively, depending on the plant species (Gorshkova et al., 2004; Chernova et al., 2018; Behr et al., 2019). The binding of LM5 to the thickened cell walls of xylem fibers was detected on the pulling side of the inclined flax (Figure 2). As noted previously, the initial deposition of a relatively loose Gn-layer and its gradual transformation into a denser G-layer is a typical developmental pattern for tertiary cell-walled cells (Gorshkova et al., 2004; Mikshina et al., 2013; Chernova et al., 2018; Gierlinger, 2018). The thickened cell walls in the flax pulling-side xylem had a similar combination of G and Gn layers (Figure 3A). Moreover, the elasticity moduli of these layers (Figure 3B) were the same as those found for flax phloem fibers using AFM (Goudenhooft et al., 2018).

Transcriptome analysis provided further evidence of the tertiary nature of thickened cell walls deposited by flax xylem in the gravitropic reaction. When compared with the opposite side and the control, in pulling-side xylem, four genes encoding FLAs and three genes encoding GT106 family members homologous to *Arabidopsis* and *Marchantia* RG-I:rhamnosyltransferases were significantly and continuously upregulated (Takenaka et al., 2018; Wachananawat et al., 2020; Table 1 and Figure 4). The importance of RG-I metabolism for tertiary cell wall formation was noted previously, and FLAs are well-known components of G-layers in tension wood (Lafarguette et al., 2004; Gerttula et al., 2015). Mokshina et al. (2020) previously described some of these genes as specifically expressed during the formation of the tertiary cell wall in flax phloem fibers in normal plant development. The four flax genes encoding FLAs, two genes encoding GT106 family members (*Lus10007975*, *Lus10013503*), and *Lus10006323* (the cytochrome P450, CYP76C6) were homologous to *Populus* genes (*Potri.004G210600*, *Potri.015G013300*, *Potri.015G048100*, and *Potri.001G109300*), which were specifically expressed in gravistimulated poplar developing G-layers (Gerttula et al., 2015), indicating the similarity of the processes taking place in these two species.

The thickening of gelatinous layers was faster than that of secondary layers in tilted poplar (Abedini et al., 2015). Similarly, the rate of tertiary cell wall deposition on the pulling side of gravistimulated flax xylem was higher than that of secondary cell walls (Figure 3B). Xylem fibers that produced tertiary cell walls deposited approximately 3.5 μm of its thickness (Gn + G), whereas cells that continued to develop the secondary cell wall layer accumulated only 0.4 μm of its thickness.

The presence of rhamnogalacturonan epitopes, similar morphology and mechanical properties of thickened cell walls in pulling-side xylem and control phloem, high rate of cell wall deposition, and coincidental gene sets expressed in gravistimulated xylem of flax, poplar tension wood, and flax bast fibers indicate that pulling-side xylem fibers deposit tertiary (gelatinous) cell walls.

### Xylem of Gravistimulated Flax Resembles Tension Wood of Arborescent Eudicots

There are few reports on the formation of reaction tissue in herbaceous plants. Tension wood can be induced in *Arabidopsis* grown under short-day and high-illumination conditions and with repeated clipping of inflorescence stems (Wyatt et al., 2010). This treatment leads to an increase in secondary xylem formation, including those inflorescence stems that are allowed to develop later. Decapitation and gravistimulation of such inflorescence stems induce tension wood formation (Wyatt et al., 2010). However, several attempts to reproduce these results failed (N. Mokshina and S. Lev-Yadun, personal communications).

Reaction tissue with gelatinous fibers is reported in both phloem and xylem of alfalfa internodes (Patten et al., 2007). However, the reaction nature of those G-fibers is doubtful, because stimulus was not applied to those plants to develop the reaction, and the G-layered tissues often were located around the stem circumference, breaking the rule of asymmetrical deposition for reaction wood (Fahn, 1990). In other reports of G-fibers in stems or tendrils of herbaceous plants (Meloche et al., 2007; Bowling and Vaughn, 2009), the term “gelatinous fibers” is not defined, and it is used for xylem fibers that are lignified with high abundance of xylans throughout the thickened cell walls, i.e., demonstrating the characteristics of normal secondary cell walls.

Thus, the gelatinous fibers developing within flax xylem with gravistimulation are one of the most reliable examples of tension wood formation in herbaceous plants. The possible reason for the reaction is the constitutive tension developed by bast fibers around the circumference of the stem (Alméras et al., 2020). This mechanism may allow for vertical orientation of the flax stem at an extremely low diameter-to-height ratio (Goudenhooft et al., 2019a). However, under gravistimulation, tension on the pulling side should increase, whereas it should decrease correspondingly on the opposite side to reorient the stem. The massive rearrangements observed in flax phloem fibers on transcriptional (Gorshkov et al., 2018), morphological (Ibragimova et al., 2017), and biochemical (Ibragimova et al., 2020) levels most likely indicate attempts to achieve that goal. The formation of tension wood in the xylem on the pulling side of the stem might also facilitate that effect.

### Comparison of Transcriptomes in Fibers With Inducible and Constitutive Tertiary Cell Wall Deposition Helps to Reveal Important Molecular Players

The mechanisms responsible for the distinct composition and architecture of tertiary walls are of increasing interest. Many attempts have been made to characterize the genes with expression activated in xylem fibers at the induction of G-layers in the formation of tension wood (Paux et al., 2005; Andersson-Gunnerås et al., 2006; Gerttula et al., 2015; Mizrachi et al., 2015). The major obstacle in such studies is the impossibility of separating xylem fibers from other xylem tissues. However, comparisons of transcriptomes in different stem parts during certain stages of bast fiber development in several species of fiber crops provide important information (Roach and Deyholos, 2007; Guerriero et al., 2017; Xie et al., 2020). Unfortunately, different tissues also complicate this approach, and therefore, differences cannot be attributed solely to gelatinous fibers. In flax, phloem fibers are at an advanced stage of specialization when they form tertiary cell walls, and because they can be quickly separated from other tissues, the transcriptome can be analyzed in a specific cell type at a defined stage of development (Gorshkov et al., 2017). Because the immunochemistry and AFM results indicated that the tertiary cell walls in phloem and xylem fibers had basic similarities, the transcriptomes of samples that contained these fibers could be compared to reveal important molecular players involved in both inducible and constitutive tertiary cell wall deposition.

In such a comparison, based on strict thresholds, ten genes were continuously upregulated in the xylem of the pulling stem side compared with the opposite side and non-inclined plants (Table 1). The genes potentially involved in the formation of RG-I are of special interest, because it is currently the least understood of the major cell wall polymers. The mechanisms of its biosynthesis and the involved glycosyltransferases remain largely unknown (Amos and Mohnen, 2019). The induction of RG-I deposition in xylem fibers (Figure 2) was coupled to the activated expression of several genes encoding enzymes from the GT106 family (Table 1). Four upregulated genes belonged to two different branches of the large *RRT* clade (Figure 4), with one containing the homologs of *RRT1* and the other those of *FRB1*. The same set of genes was expressed in all samples of phloem fibers. The enzymes encoded by homologs of both *RRT1* (Takenaka et al., 2018) and *FRB1* (Wachananawat et al., 2020) provide the same linkage in the RG-I backbone by adding rhamnosyl residue to the preceding galacturonosyl residue. The expression of the same four rhamnosyltransferase genes from GT106 in all samples that contained fibers depositing the tertiary cell wall but not in others (Table 1) suggests that the encoded enzymes are associated with the biosynthesis of RG-I, which is a characteristic component of tertiary cell walls (Gorshkova et al., 2018). Activation of RG-I biosynthesis in the phloem fibers on the pulling side of the plant, when compared with control plants, was suggested previously on the basis of biochemical and immunochemical data (Ibragimova et al., 2020). The results in the current study are consistent with those results, and all four GT106 genes that were activated in xylem of the pulling side were also upregulated in phloem fibers on the same stem side (Table 1).

The simultaneous expression of four different genes from the *RRT* clade in isolated phloem fibers indicated a complex consisting of similar but not identical proteins that might be involved in the formation of the RG-I backbone. The necessity of several proteins encoded by the members of the same gene family has been previously established for the biosynthesis of homogalacturonan (Atmodjo et al., 2011), xylan (Zeng et al., 2010), and cellulose (Desprez et al., 2007). A similar situation was recently suggested for *MpRRT1-4* genes by Professor Ishimizu’s group based on analysis of expression in various organs of the liverwort *Marchantia polymorpha* (Wachananawat et al., 2020).

Rhamnogalacturonan I is a complex polysaccharide that contains several different monosaccharides linked by numerous linkage types. However, upregulation was detected only for the genes of the enzymes that provided a specific linkage within the backbone (Table 1). This result could be explained by the poor identification of glycosyltransferases involved in RG-I biosynthesis (Amos and Mohnen, 2019). However, the flax homologs of *At5g44670* that encodes the galactosyltransferase GALS1, which likely participates in formation of galactan side chains attached to the RG-I backbone (Liwanag et al., 2012), were not detected among those constitutively upregulated at induction of the G-layer, despite pronounced biosynthesis of galactan revealed by the LM5 antibody (Figure 2). Although their expression was pronounced, it was similar in xylem of pulling and opposite stem sides (data not shown). Collectively, these data suggest the formation of the complex that makes the RG-I backbone is the limiting step in the biosynthesis of this polymer.

Overall, the comparison of transcriptomes in fibers with inducible and constitutive tertiary cell wall deposition was effective in revealing important molecular players involved in the formation of cellulose-enriched cell walls. Further analysis of this system and extending the time scale of experiments to earlier stages of gravitropic response may provide clues to better understand the mechanisms of G-layer deposition and the regulation of cell wall formation in general.

## Conclusion

Phloem fibers of many fiber crops form tertiary cell walls constitutively, while in xylem fibers of dicotyledonous trees the deposition of such walls can be induced by gravistimulation in so-called tension wood. Flax is the only unique example of herbaceous plant where both constitutive and inducible tertiary cell wall formation is possible. Using AFM, immunochemistry, and transcriptomic analysis, we have shown that these cell walls are similar in their immunogenicity and mechanical properties and recruit the same subset of genes during formation.

## Data Availability Statement

The datasets presented in this study can be found in online repositories. The names of the repository and accession number can be found below: https://www.ncbi.nlm.nih.gov/, PRJNA631357.

## Author Contributions

AP, LK, and TG: conceptualization. AP, LK, AN, MA, and OG: investigation. AP, LK, and MA: methodology. AP, AN, and OG: visualization. AP, OG, LK, and TG: writing—original draft. LK and TG: writing—review and editing. All authors contributed to the article and approved the submitted version.

## Conflict of Interest

The authors declare that the research was conducted in the absence of any commercial or financial relationships that could be construed as a potential conflict of interest.

## Footnotes

1 https://imagej.net/

2 https://www.ncbi.nlm.nih.gov/sra

3 https://phytozome.jgi.doe.gov/

4 http://hmmer.org/

5 http://pfam.xfam.org/

6 https://phytozome.jgi.doe.gov/pz/portal.html

7 https://www.ebi.ac.uk/interpro/

8 https://www.uniprot.org/

9 https://www.ebi.ac.uk/Tools/msa/clustalo/

10 https://itol.embl.de/

## References

[B1] Abedini R. Clair B. Pourtahmasi K. Laurans F. Arnould O. (2015). Cell Wall thickening in developing tension wood of artificially bent poplar trees. *IAWA J.* 36 44–57. 10.1163/22941932-00000084

[B2] Alméras T. Petrova A. Kozlova L. Gril J. Gorshkova T. (2020). Evidence and quantitative evaluation of tensile maturation strain in flax phloem through longitudinal splitting. *Botany* 98 9–19. 10.1139/cjb-2019-0021

[B3] Amos R. A. Mohnen D. (2019). Critical review of plant cell wall matrix polysaccharide glycosyltransferase activities verified by heterologous protein expression. *Front. Plant Sci.* 10:915. 10.3389/fpls.2019.00915 31379900 PMC6646851

[B4] Andersson-Gunnerås S. Mellerowicz E. J. Love J. Segerman B. Ohmiya Y. Coutinho P. M. (2006). Biosynthesis of cellulose-enriched tension wood in *Populus*: global analysis of transcripts and metabolites identifies biochemical and developmental regulators in secondary wall biosynthesis. *Plant J.* 45 144–165. 10.1111/j.1365-313X.2005.02584.x 16367961

[B5] Arnould O. Siniscalco D. Bourmaud A. Le Duigou A. Baley C. (2017). Better insight into the nano-mechanical properties of flax fibre cell walls. *Ind. Crops Prod.* 97 224–228. 10.1016/j.indcrop.2016.12.020

[B6] Atmodjo M. A. Sakuragi Y. Zhu X. Burrell A. J. Mohanty S. S. Atwood J. A. (2011). Galacturonosyltransferase (GAUT)1 and GAUT7 are the core of a plant cell wall pectin biosynthetic homogalacturonan: galacturonosyltransferase complex. *Proc. Natl. Acad. Sci. U.S.A.* 108 20225–20230. 10.1073/pnas.1112816108 22135470 PMC3250160

[B7] Bateman A. Martin M. J. Orchard S. Magrane M. Alpi E. Bely B. (2019). UniProt: a worldwide hub of protein knowledge. *Nucleic Acids Res.* 47 D506–D515. 10.1093/nar/gky1049 30395287 PMC6323992

[B8] Behr M. Faleri C. Hausman J. F. Planchon S. Renaut J. Cai G. (2019). Distribution of cell-wall polysaccharides and proteins during growth of the hemp hypocotyl. *Planta* 250 1539–1556. 10.1007/s00425-019-03245-9 31352512

[B9] Bobich E. G. Nobel P. S. (2002). Cladode junction regions and their biomechanics for arborescent *Platyopuntias*. *Int. J. Plant Sci.* 163 507–517. 10.1086/340443

[B10] Bourmaud A. Beaugrand J. Shah D. U. Placet V. Baley C. (2018). Towards the design of high-performance plant fibre composites. *Prog. Mater. Sci.* 97 347–408. 10.1016/j.pmatsci.2018.05.005

[B11] Bowling A. J. Vaughn K. C. (2009). Gelatinous fibers are widespread in coiling tendrils and twining vines. *Am. J. Bot.* 96 719–727. 10.3732/ajb.0800373 21628227

[B12] Camacho C. Coulouris G. Avagyan V. Ma N. Papadopoulos J. Bealer K. (2009). BLAST plus: architecture and applications. *BMC Bioinform.* 10:421. 10.1186/1471-2105-10-421 20003500 PMC2803857

[B13] Chernova T. Ageeva M. Mikshina P. Trofimova O. Kozlova L. Lev-Yadun S. (2020). The living fossil *Psilotum nudum* has cortical fibers with mannan-based cell wall matrix. *Front. Plant Sci.* 11:488. 10.3389/fpls.2020.00488 32411161 PMC7199214

[B14] Chernova T. E. Mikshina P. V. Salnikov V. V. Ibragimova N. N. Sautkina O. V. Gorshkova T. A. (2018). Development of distinct cell wall layers both in primary and secondary phloem fibers of hemp (*Cannabis sativa* L.). *Ind. Crops Prod.* 117 97–109. 10.1016/j.indcrop.2018.02.082

[B15] Clair B. Alméras T. Pilate G. Jullien D. Sugiyama J. Riekel C. (2011). Maturation stress generation in poplar tension wood studied by synchrotron radiation microdiffraction. *Plant Physiol.* 155 562–570. 10.1104/pp.110.167270 21068364 PMC3075793

[B16] Clair B. Dejardin A. Pilate G. Alméras T. (2018). Is the G-Layer a tertiary cell wall? *Front. Plant Sci.* 9:623. 10.3389/fpls.2018.00623 29868079 PMC5952352

[B17] Coste R. Pernes M. Tetard L. Molinari M. Chabbert B. (2020). Effect of the interplay of composition and environmental humidity on the nanomechanical properties of hemp fibers. *ACS Sust. Chem. Eng.* 8 6381–6390. 10.1021/acssuschemeng.0c00566

[B18] Côté W. A. Day A. C. Timell T. E. (1969). A contribution to the ultrastructure of tension wood fibers. *Wood Sci. Technol.* 3 257–271. 10.1007/BF00352301

[B19] Desprez T. Juraniec M. Crowell E. F. Jouy H. Pochylova Z. Parcy F. (2007). Organization of cellulose synthase complexes involved in primary cell wall synthesis in *Arabidopsis thaliana*. *Proc. Natl. Acad. Sci. U.S.A.* 104 15572–15577. 10.1073/pnas.0706569104 17878303 PMC2000492

[B20] El-Gebali S. Mistry J. Bateman A. Eddy S. R. Luciani A. Potter S. C. (2019). The Pfam protein families database in 2019. *Nucleic Acids Res.* 47 D427–D432. 10.1093/nar/gky995 30357350 PMC6324024

[B21] Esau K. (1965). *Plant Anatomy.* New York, NY: John Wiley & Sons.

[B22] Fahn A. (1990). *Plant Anatomy.* Oxford: Pergamon Press.

[B23] Fisher J. B. (2008). Anatomy of axis contraction in seedlings from a fire prone habitat. *Am. J. Bot.* 95 1337–1348. 10.3732/ajb.0800083 21628143

[B24] Fisher J. B. Blanco M. A. (2014). Gelatinous fibers and variant secondary growth related to stem undulation and contraction in a monkey ladder vine, *Bauhinia glabra* (*Fabaceae*). *Am. J. Bot.* 101 608–616. 10.3732/ajb.1300407 24699542

[B25] Gerttula S. Zinkgraf M. Muday G. K. Lewis D. R. Ibatullin F. M. Brumer H. (2015). Transcriptional and hormonal regulation of gravitropism of woody stems in *Populus*. *Plant Cell* 27 2800–2813. 10.1105/tpc.15.00531 26410302 PMC4682325

[B26] Gierlinger N. (2018). New insights into plant cell walls by vibrational microspectroscopy. *Appl. Spectrosc. Rev.* 53 517–551. 10.1080/05704928.2017.1363052 30057488 PMC6050719

[B27] Goodstein D. M. Shu S. Q. Howson R. Neupane R. Hayes R. D. Fazo J. (2012). Phytozome: a comparative platform for green plant genomics. *Nucleic Acids Res.* 40 D1178–D1186. 10.1093/nar/gkr944 22110026 PMC3245001

[B28] Gorshkov O. Mokshina N. Gorshkov V. Chemikosova S. Gogolev Y. Gorshkova T. (2017). Transcriptome portrait of cellulose-enriched flax fibres at advanced stage of specialization. *Plant Mol. Biol.* 93 431–449. 10.1007/s11103-016-0571-7 27981388

[B29] Gorshkov O. Mokshina N. Ibragimova N. Ageeva M. Gogoleva N. Gorshkova T. (2018). Phloem fibres as motors of gravitropic behaviour of flax plants: level of transcriptome. *Funct. Plant Biol.* 45 203–214. 10.1071/Fp16348 32291034

[B30] Gorshkova T. Brutch N. Chabbert B. Deyholos M. Hayashi T. Lev-Yadun S. (2012). Plant fiber formation: state of the art, recent and expected progress, and open questions. *Crit. Rev. Plant Sci.* 31 201–228. 10.1080/07352689.2011.616096

[B31] Gorshkova T. Chernova T. Mokshina N. Ageeva M. Mikshina P. (2018). Plant ‘muscles’: fibers with a tertiary cell wall. *New Phytol.* 218 66–72. 10.1111/nph.14997 29364532

[B32] Gorshkova T. Mokshina N. Chernova T. Ibragimova N. Salnikov V. Mikshina P. (2015). Aspen tension wood fibers contain beta-(1 -> 4)-galactans and acidic arabinogalactans retained by cellulose microfibrils in gelatinous walls. *Plant Physiol.* 169 2048–2063. 10.1104/pp.15.00690 26378099 PMC4634055

[B33] Gorshkova T. A. Chemikosova S. B. Sal’nikov V. V. Pavlencheva N. V. Gur’janov O. P. Stolle-Smits T. (2004). Occurrence of cell-specific galactan is coinciding with bast fiber developmental transition in flax. *Ind. Crops Prod.* 19 217–224. 10.1016/j.indcrop.2003.10.002

[B34] Gorshkova T. A. Gurjanov O. P. Mikshina P. V. Ibragimova N. N. Mokshina N. E. Salnikov V. V. (2010). Specific type of secondary cell wall formed by plant fibers. *Russ. J. Plant Physl.* 57 328–341. 10.1134/S1021443710030040

[B35] Goudenhooft C. Bourmaud A. Baley C. (2019a). Flax (*Linum usitatissimum* L.) fibers for composite reinforcement: exploring the link between plant growth, cell walls development, and fiber properties. *Front. Plant Sci.* 10:411. 10.3389/fpls.2019.00411 31001310 PMC6456768

[B36] Goudenhooft C. Bourmaud A. Baley C. (2019b). Study of plant gravitropic response: exploring the influence of lodging and recovery on the mechanical performances of flax fibers. *Ind. Crops Prod.* 128 235–238. 10.1016/j.indcrop.2018.11.024

[B37] Goudenhooft C. Siniscalco D. Arnould O. Bourmaud A. Sire O. Gorshkova T. (2018). Investigation of the mechanical properties of flax cell walls during plant development: the relation between performance and cell wall structure. *Fibers* 6:6. 10.3390/fib6010006

[B38] Gritsch C. Wan Y. F. Mitchell R. A. C. Shewry P. R. Hanley S. J. Karp A. (2015). G-fibre cell wall development in willow stems during tension wood induction. *J. Exp. Bot.* 66 6447–6459. 10.1093/jxb/erv358 26220085 PMC4588891

[B39] Groover A. (2016). Gravitropisms and reaction woods of forest trees - evolution, functions and mechanisms. *New Phytol.* 211 790–802. 10.1111/nph.13968 27111862

[B40] Guedes F. T. P. Laurans F. Quemener B. Assor C. Laine-Prade V. Boizot N. (2017). Non-cellulosic polysaccharide distribution during G-layer formation in poplar tension wood fibers: abundance of rhamnogalacturonan I and arabinogalactan proteins but no evidence of xyloglucan. *Planta* 246 857–878. 10.1007/s00425-017-2737-1 28699115

[B41] Guerriero G. Behr M. Legay S. Mangeot-Peter L. Zorzan S. Ghoniem M. (2017). Transcriptomic profiling of hemp bast fibres at different developmental stages. *Sci. Rep.* 7:4961. 10.1038/s41598-017-05200-8 28694530 PMC5504027

[B42] Hall T. A. (1999). BioEdit: a user-friendly biological sequence alignment editor and analysis program for Windows 95/98/NT. *Nucleic Acids Symp. Ser.* 41 95–98.

[B43] Ibragimova N. Mokshina N. Ageeva M. Gurjanov O. Mikshina P. (2020). Rearrangement of the cellulose-enriched cell wall in flax phloem fibers over the course of the gravitropic reaction. *Int. J. Mol. Sci.* 21:5322. 10.3390/ijms21155322 32727025 PMC7432630

[B44] Ibragimova N. N. Ageeva M. V. Gorshkova T. A. (2017). Development of gravitropic response: unusual behavior of flax phloem G-fibers. *Protoplasma* 254 749–762. 10.1007/s00709-016-0985-8 27263083

[B45] Kaku T. Serada S. Baba K. Tanaka F. Hayashi T. (2009). Proteomic analysis of the G-layer in poplar tension wood. *J. Wood Sci.* 55 250–257. 10.1007/s10086-009-1032-6

[B46] Kalyaanamoorthy S. Minh B. Q. Wong T. K. F. von Haeseler A. Jermiin L. S. (2017). ModelFinder: fast model selection for accurate phylogenetic estimates. *Nat. Methods* 14 587–589. 10.1038/Nmeth.4285 28481363 PMC5453245

[B47] Lafarguette F. Leple J. C. Dejardin A. Laurans F. Costa G. Lesage-Descauses M. C. (2004). Poplar genes encoding fasciclin-like arabinogalactan proteins are highly expressed in tension wood. *New Phytol.* 164 107–121. 10.1111/j.1469-8137.2004.01175.x 33873473

[B48] Letunic I. Bork P. (2019). Interactive Tree Of Life (iTOL) v4: recent updates and new developments. *Nucleic Acids Res.* 47 W256–W259. 10.1093/nar/gkz239 30931475 PMC6602468

[B49] Liwanag A. J. M. Ebert B. Verhertbruggen Y. Rennie E. A. Rautengarten C. Oikawa A. (2012). Pectin biosynthesis: GALS1 in *Arabidopsis thaliana* is a beta-1,4-galactan beta-1,4-galactosyltransferase. *Plant Cell* 24 5024–5036. 10.1105/tpc.112.106625 23243126 PMC3556973

[B50] Love G. D. Snape C. E. Jarvis M. C. Morrison I. M. (1994). Determination of phenolic structures in flax fiber by solid-state C-13 NMR. *Phytochemistry* 35 489–491. 10.1016/S0031-9422(00)94788-5

[B51] Love M. I. Anders S. Kim V. Huber W. (2015). RNA-Seq workflow: gene-level exploratory analysis and differential expression. *F1000Research* 4:1070. 10.12688/f1000research.7035.1 26674615 PMC4670015

[B52] Madeira F. Park Y. M. Lee J. Buso N. Gur T. Madhusoodanan N. (2019). The EMBL-EBI search and sequence analysis tools APIs in 2019. *Nucleic Acids Res.* 47 W636–W641. 10.1093/nar/gkz268 30976793 PMC6602479

[B53] McCartney L. Marcus S. E. Knox J. P. (2005). Monoclonal antibodies to plant cell wall xylans and arabinoxylans. *J. Histochem. Cytochem.* 53 543–546. 10.1369/jhc.4B6578.2005 15805428

[B54] Meloche C. G. Knox J. P. Vaughn K. C. (2007). A cortical band of gelatinous fibers causes the coiling of redvine tendrils: a model based upon cytochemical and immunocytochemical studies. *Planta* 225 485–498. 10.1007/s00425-006-0363-4 16955273

[B55] Mikshina P. Chernova T. Chemikosova S. Ibragimova N. Mokshina N. Gorshkova T. (2013). “Cellulosic fibers: role of matrix polysaccharides in structure and function,” in *Cellulose - Fundamental Aspects*, eds van de Ven T. Godbout L. (London: IntechOpen), 91–112. 10.5772/51941

[B56] Minh B. Q. Nguyen M. A. T. von Haeseler A. (2013). Ultrafast approximation for phylogenetic bootstrap. *Mol. Biol. Evol.* 30 1188–1195. 10.1093/molbev/mst024 23418397 PMC3670741

[B57] Mitchell A. L. Attwood T. K. Babbitt P. C. Blum M. Bork P. Bridge A. (2019). InterPro in 2019: improving coverage, classification and access to protein sequence annotations. *Nucleic Acids Res.* 47 D351–D360. 10.1093/nar/gky1100 30398656 PMC6323941

[B58] Mizrachi E. Maloney V. J. Silberbauer J. Hefer C. A. Berger D. K. Mansfield S. D. (2015). Investigating the molecular underpinnings underlying morphology and changes in carbon partitioning during tension wood formation in *Eucalyptus*. *New Phytol.* 206 1351–1363. 10.1111/nph.13152 25388807

[B59] Mokshina N. Chernova T. Galinousky D. Gorshkov O. Gorshkova T. (2018). Key stages of fiber development as determinants of bast fiber yield and quality. *Fibers* 6:20. 10.3390/fib6020020

[B60] Mokshina N. Gorshkov O. Galinousky D. Gorshkova T. (2020). Genes with bast fiber-specific expression in flax plants - Molecular keys for targeted fiber crop improvement. *Ind. Crops Prod.* 152:112549. 10.1016/j.indcrop.2020.112549

[B61] Mokshina N. Gorshkov O. Ibragimova N. Chernova T. Gorshkova T. (2017). Cellulosic fibres of flax recruit both primary and secondary cell wall cellulose synthases during deposition of thick tertiary cell walls and in the course of graviresponse. *Funct. Plant Biol.* 44 820–831. 10.1071/Fp17105 32480610

[B62] Nguyen L. T. Schmidt H. A. von Haeseler A. Minh B. Q. (2015). IQ-TREE: a fast and effective stochastic algorithm for estimating maximum-likelihood phylogenies. *Mol. Biol. Evol.* 32 268–274. 10.1093/molbev/msu300 25371430 PMC4271533

[B63] Norberg P. H. Meier H. (1966). Physical and chemical properties of the gelatinous layer in tension wood fibres of aspen (*Populus tremula* L.). *Holzforschung* 20 174–178. 10.1515/hfsg.1966.20.6.174

[B64] Patten A. M. Jourdes M. Brown E. E. Laborie M. P. Davin L. B. Lewis N. G. (2007). Reaction tissue formation and stem tensile modulus properties in wild-type and p-coumarate-3-hydroxylase downregulated lines of alfalfa, *Medicago sativa* (*Fabaceae*). *Am. J. Bot.* 94 912–925. 10.3732/ajb.94.6.912 21636460

[B65] Paux E. Carocha V. Marques C. de Sousa A. M. Borralho N. Sivadon P. (2005). Transcript profiling of Eucalyptus xylem genes during tension wood formation. *New Phytol.* 167 89–100. 10.1111/j.1469-8137.2005.01396.x 15948833

[B66] Pertea M. Kim D. Pertea G. M. Leek J. T. Salzberg S. L. (2016). Transcript-level expression analysis of RNA-seq experiments with HISAT, StringTie and Ballgown. *Nat. Prot.* 11 1650–1667. 10.1038/nprot.2016.095 27560171 PMC5032908

[B67] Roach M. J. Deyholos M. K. (2007). Microarray analysis of flax (*Linum usitatissimum* L.) stems identifies transcripts enriched in fibre-bearing phloem tissues. *Mol. Genet. Genom.* 278 149–165. 10.1007/s00438-007-0241-1 17503083

[B68] Roussel J. R. Clair B. (2015). Evidence of the late lignification of the G-layer in *Simarouba* tension wood, to assist understanding how non-G-layer species produce tensile stress. *Tree Physiol.* 35 1366–1377. 10.1093/treephys/tpv082 26427915

[B69] Schreiber N. Gierlinger N. Putz N. Fratzl P. Neinhuis C. Burgert I. (2010). G-fibres in storage roots of *Trifolium pratense* (*Fabaceae*): tensile stress generators for contraction. *Plant J.* 61 854–861. 10.1111/j.1365-313X.2009.04115.x 20030750

[B70] SEQC/MAQC-III Consortium (2014). A comprehensive assessment of RNA-seq accuracy, reproducibility and information content by the sequencing quality control consortium. *Nat. Biotechnol.* 32, 903–914. 10.1038/nbt.2957 25150838 PMC4321899

[B71] Takenaka Y. Kato K. Ogawa-Ohnishi M. Tsuruhama K. Kajiura H. Yagyu K. (2018). Pectin RG-I rhamnosyltransferases represent a novel plant-specific glycosyltransferase family. *Nat. Plants* 4 669–676. 10.1038/s41477-018-0217-7 30082766

[B72] Tomlinson P. B. Magellan T. M. Griffith M. P. (2014). Root contraction in *Cycas* and *Zamia* (*Cycadales*) determined by gelatinous fibers. *Am. J. Bot.* 101 1275–1285. 10.3732/ajb.1400170 25077507

[B73] Wachananawat B. Kuroha T. Takenaka Y. Kajiura H. Naramoto S. Yokoyama R. (2020). Diversity of pectin rhamnogalacturonan I rhamnosyltransferases in glycosyltransferase family 106. *Front. Plant Sci.* 11:997. 10.3389/fpls.2020.00997 32714362 PMC7343896

[B74] Wang Z. W. Hobson N. Galindo L. Zhu S. L. Shi D. H. McDill J. (2012). The genome of flax (*Linum usitatissimum*) assembled *de novo* from short shotgun sequence reads. *Plant J.* 72 461–473. 10.1111/j.1365-313X.2012.05093.x 22757964

[B75] Wyatt S. E. Sederoff R. Flaishman M. A. Lev-Yadun S. (2010). Arabidopsis thaliana as a model for gelatinous fiber formation. *Russ. J. Plant Phys.* 57 363–367. 10.1134/S1021443710030076

[B76] Xie J. Y. Li J. Q. Jie Y. C. Xie D. Y. Yang D. Shi H. Z. (2020). Comparative transcriptomics of stem bark reveals genes associated with bast fiber development in *Boehmeria nivea* L. gaud (ramie). *BMC Genom.* 21:40. 10.1186/s12864-020-6457-8 31931705 PMC6958601

[B77] Zeng W. Jiang N. Nadella R. Killen T. L. Nadella V. Faik A. (2010). A Glucurono(arabino)xylan synthase complex from wheat contains members of the GT43, GT47, and GT75 families and functions cooperatively. *Plant Physiol.* 154 78–97. 10.1104/pp.110.159749 20631319 PMC2938142

[B78] Zimmermann M. H. Wardrop A. B. Tomlinson P. B. (1968). Tension wood in aerial roots of *Ficus benjamina* L. *Wood Sci. Technol.* 2 95–104. 10.1007/BF00394958

